# Identification of nonlinear effects of background asymmetry on solitary oscillations in a cylindrical plasma

**DOI:** 10.1038/s41598-024-62969-1

**Published:** 2024-05-28

**Authors:** Taiki Kobayashi, Akihide Fujisawa, Yoshihiko Nagashima, Chanho Moon, Kotaro Yamasaki, Daiki Nishimura, Sigeru Inagaki, Akihiro Shimizu, Tokihiko Tokuzawa, Takeshi Ido

**Affiliations:** 1 Interdisciplinary Graduate School of Engineering Sciences, Kyushu University, Kasuga, 816-8580 Japan; 2 Research Institute for Applied Mechanics, Kyushu University, Kasuga, 816-8580 Japan; 3 Research Center for Plasma Turbulence, Kyushu University, Kasuga, 816-8580 Japan; 4 National Institute for Fusion Science, National Institute of Natural Sciences, Toki, 509-5292 Japan; 5 Graduate School of Advanced Science and Engineering, Hiroshima University, Higashihiroshima, 739-8527 Japan; 6 Institute of Advanced Energy, Kyoto University, Uji, 611-0011 Japan

**Keywords:** Plasma turbulence, Symmetry breaking, Tomography, Magnetically confined plasmas, Imaging and sensing

## Abstract

A symmetry-breaking in rotational spatial pattern of quasi-periodic solitary oscillations is revealed with tomography measurement of plasma emission, simultaneously with background asymmetry in stationary plasma structure. Although the oscillatory pattern deformation is a natural course in the presence of asymmetry, elaborate analyses identify existence unfeatured nonlinear effects of the background asymmetry, i.e., its nonlinear couplings with harmonic modes of rotational symmetry, to produce non-harmonic mode to break the symmetry and cause the oscillatory pattern to be chaotic. The findings suggest the unrecognized fundamental process for plasmas to be turbulent.

## Introduction

Symmetry is one of the fundamental concepts to constrain the physical systems through conservation of physical quantity such as angular momentum, momentum and energy. The concept is also important for structural formation and dynamics of plasma turbulence. In research of magnetic confinement plasmas, it is observed in several experiments that symmetry breaking of turbulence field in response to inhomogeneous confinement fields^1–5^. Moreover, the research brings a recent view that interaction between fluctuations of disparate (micro-, meso- and macro-) scales sustains the plasma structure^6–10^. Along with these concepts of symmetry breaking and cross-scale coupling, the entire field measurements of plasma turbulence field are required for further understanding of plasma turbulence.

Imaging measurement is a promising candidate for such measurement, and various imaging techniques, using electron cyclotron emission, soft x-ray, visible light emission, and so on, have been carried out in toroidal and linear device^11–20^. Tomography systems have been developed to measure the entire plasma cross-section in the linear cylindrical plasma device called Plasma Assembly for Nonlinear Turbulence Analysis (PANTA)^21,22^. Recently, such measurement has revealed symmetry breaking in rotating spatial pattern, which has been considered rotationally symmetric, during cycle of so-called solitary oscillations, which is nonlinear quasi-periodic oscillation originating from drift wave instabilities^23–25^. This paper presents the spatiotemporal images obtained in the tomography measurement of the entire plasma structure and fluctuations, which provides resultant findings to pay attention to unrecognized nonlinear effects of the background asymmetry to cause plasma to become more turbulent.

## Results

**Figure 1:**
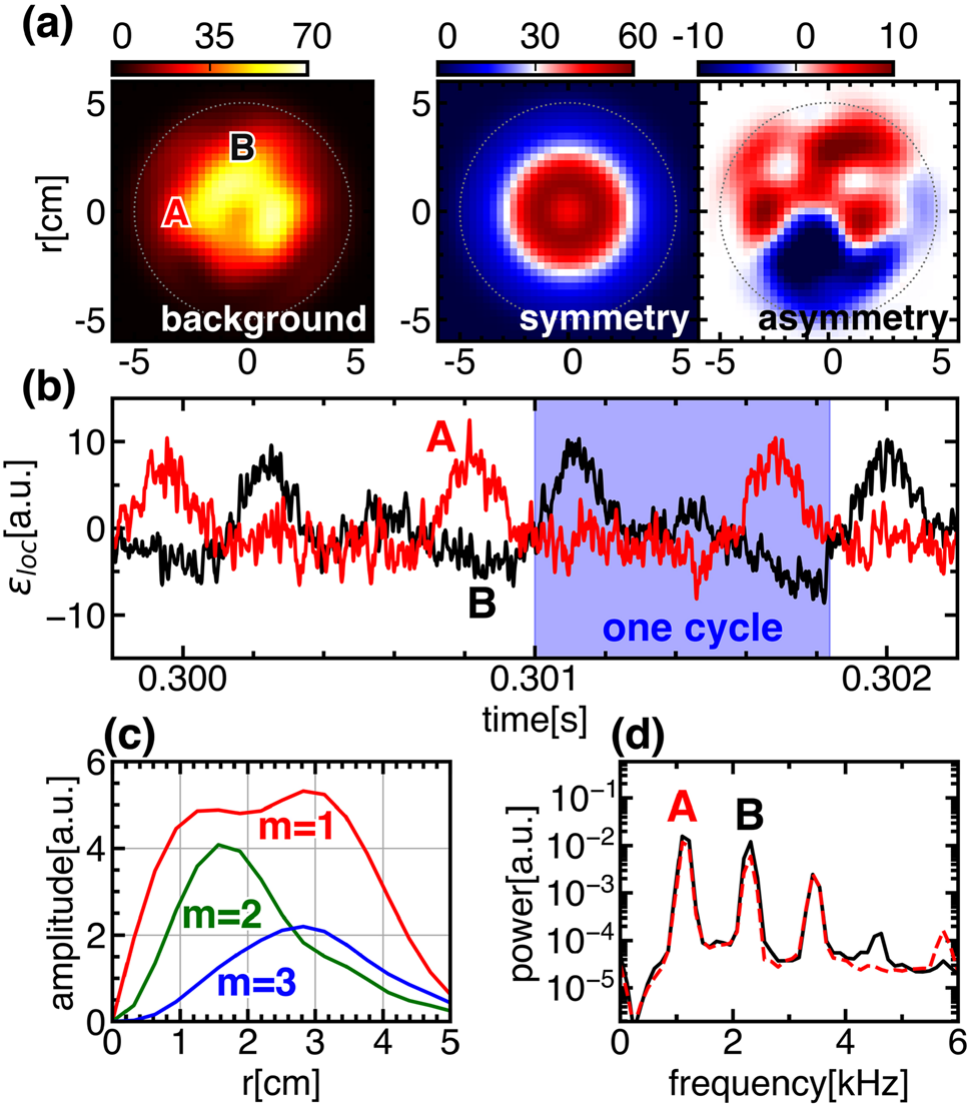
(**a**) Temporally averaged emission profile and its symmetric and asymmetric part fitted by Fourier-Bessel function series. (**b**) Temporal evolution of local emission. The red and black lines show the local positions (x, y) = (− 3 cm, 0 cm) and (0 cm, 3 cm) respectively. (**c**) The radial profile of the amplitude of each azimuthal mode in the background structure. (**d**) The frequency spectra of local emissions.

In PANTA, helicon waves heating generates argon plasma with a radius of 5 cm for 0.6 s. The filling gas pressure and magnetic field strength are set to be 2.16 mTorr and 700 G, respectively, in which condition the solitary oscillation is observed. In the tomography measurement, 128 channels of the line-integrated emissions of ArI are obtained with 1 MHz sampling. The local emission profile is reconstructed with a spatial resolution of about 1.45 cm using an algorithm called the Maximum Likelihood-Expectation Maximization (MLEM)^26^. In addition, the symmetric and asymmetric components of the structure can be obtained by Fourier-Bessel function (FBF)^27–29^ or Fourier-Rectangular function (FRF)^30^, so that the emission profile can be divided into,

$$\begin{aligned} \epsilon (r, \theta , t)=\bar{\epsilon }_{S}(r, \theta )+\bar{\epsilon }_{A}(r, \theta )+\tilde{\epsilon }_{d}(r, \theta , t)+\tilde{\epsilon }_{p}(r, \theta , t), \end{aligned}$$

where $\bar{\epsilon }_{S}$, $\bar{\epsilon }_{A}$, $\tilde{\epsilon }_{d}$ and $\tilde{\epsilon }_{p}$ represent the time-averaged (background) symmetric and asymmetric parts, and deterministic and probabilistic part of fluctuation, respectively. Figure 1 shows an overview of the target plasma. Here, the time-averaged symmetric and asymmetric parts shown in Fig. 1a are expanded using FBF as in the equation below,

1 $$\begin{aligned} \epsilon (r, \theta )&= \sum _{n}a_{0n}J_{0}(k_{0n}r)\nonumber \\&\quad + \sum _{m, n}J_{m}(k_{mn}r)(a_{mn}\cos (m\theta )+b_{mn}\sin (m\theta )). \end{aligned}$$

The symmetric and non-symmetric parts are represented by the first and second terms on the right-hand side of Eq. (1). The asymmetry part, $\bar{\epsilon }_{A}$, is reproduced with the non-symmetric terms. The asymmetry could be caused by the structural inhomogeneity of plasma production systems such as heating source, particle source, and so on. The temporal evolution of the local emissions in Fig. 1b show solitary oscillations, while the frequency spectrum shown in Fig. 1d clearly shows the presence of the fundamental frequency ($\omega \sim 1.2$ kHz) and harmonics, which are characteristic of the solitary oscillation, from which coherent spatiotemporal evolution is expected without deforming their shape.

**Figure 2:**
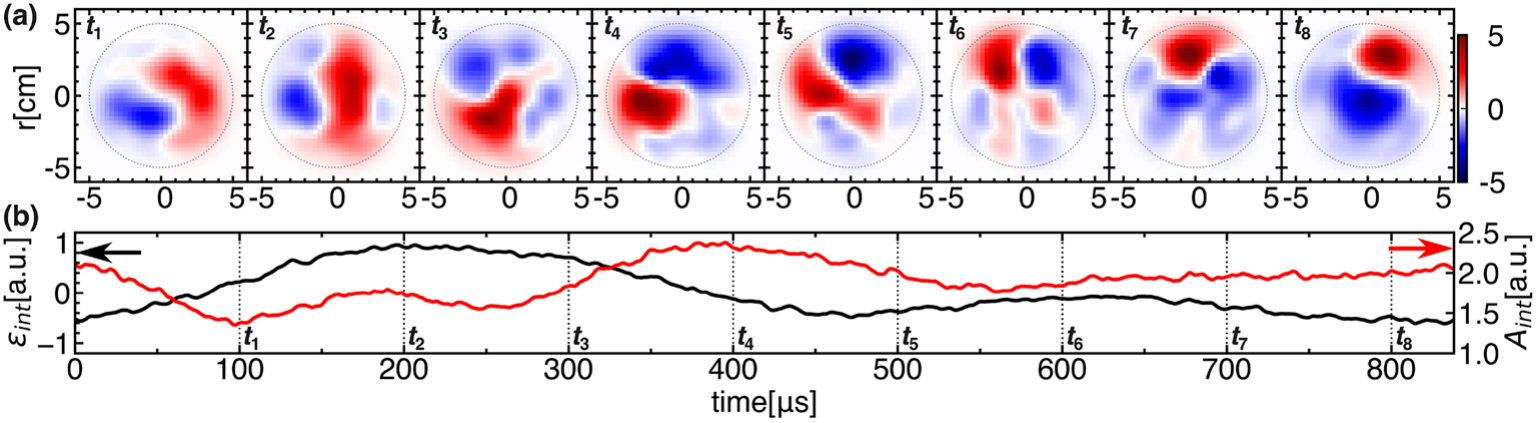
(**a**) Temporal evolution of the 2D structure of deterministic trend of solitary oscillations. (**b**) The corresponding temporal evolution of the fluctuating part of the integrated emission (black line) and its amplitude (red line).

**Figure 3:**
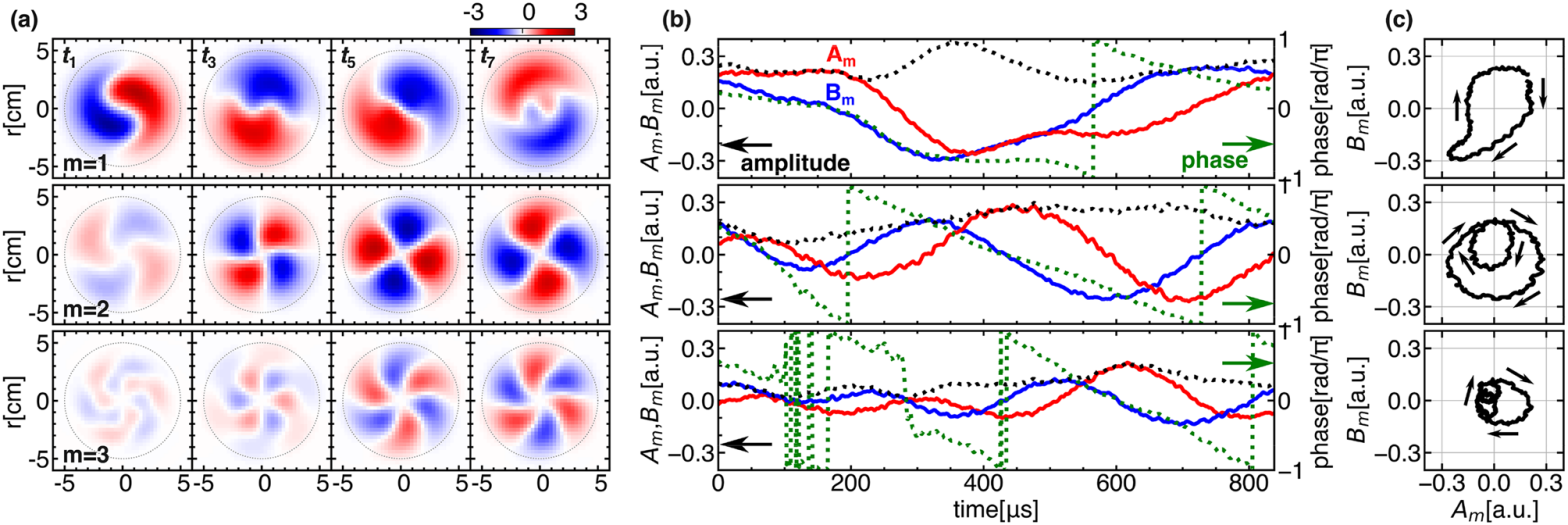
(**a**) Temporal evolution of 2D image of each azimuthal mode. (**b**) The temporal evolution of the moment vector elements $A_{m}$ and $B_{m}$ of azimuthal modes (red and blue line) with and its amplitude (black dotted line) and phase (green dotted line). (**c**) Lissajous trajectories of moment vector elements $A_{m}$ and $B_{m}$.

The deterministic trend of the solitary oscillations is obtained with an advanced method of conditional average, so called Correlation-Estimated Conditional Averaged Method (CECAME) to give a procedure to select the optimized ensemble^31^. Figure 2 shows the result of the 2D-images during a period of the solitary oscillations, with temporal evolution of its integrated emission and amplitude whose definitions are $\int \tilde{\epsilon }_{d}(r,\theta ,t)rdrd\theta$ and $\sqrt{\int \tilde{\epsilon }^2_{d}(r,\theta ,t)rdrd\theta }$. It is found that the conditionally-averaged (or deterministic) images of the oscillations are dominated with a rotating *m *= 1 azimuthal mode in the electron diamagnetic (or clockwise) direction, and suffer from spatial deformation during the period. The deformation in deterministic trend to break the rotational symmetry should never happen if the system is under axial symmetry or $\bar{\epsilon }_{A}(r,\theta )=0.$

A modal decomposition is performed to quantify the observed pattern deformation. Figure 3a shows 2D images of azimuthal modes, $m=1, 2$ and 3 after the modal decomposition using the FBF. It is obvious that each mode changes its pattern with rotation during the period. The manner of deformation can be evaluated as an azimuthal modal vector $\overrightarrow{M}(t)=(A_{m}(t), B_{m}(t))$^22^, whose elements are calculated from the 2D images as follows,

$$\begin{aligned} A_{m}(t)&= \frac{1}{\sqrt{\pi }}\int \tilde{\epsilon }(r, \theta , t)\cos (m\theta )rdrd\theta ,\\ B_{m}(t)&= \frac{1}{\sqrt{\pi }}\int \tilde{\epsilon }(r, \theta , t)\sin (m\theta )rdrd\theta . \end{aligned}$$

Note that the absolute value and phase of the modal vector represents the amplitude and location of each mode structure. Figure 3b,c also shows the temporal evolution of the vector elements, and their Lissajous trajectories. The amplitude of each azimuthal mode is found to vary during the cycle, and the corresponding Lissajous trajectories are found to deform from circles that would be expected if a mode simply rotated without deformation. This deviation of the Lissajous trajectory from a circle is ascribed to emergence of backward propagating harmonic and/or non-harmonic frequency modes which result in different phase velocities from harmonic one.

**Figure 4:**
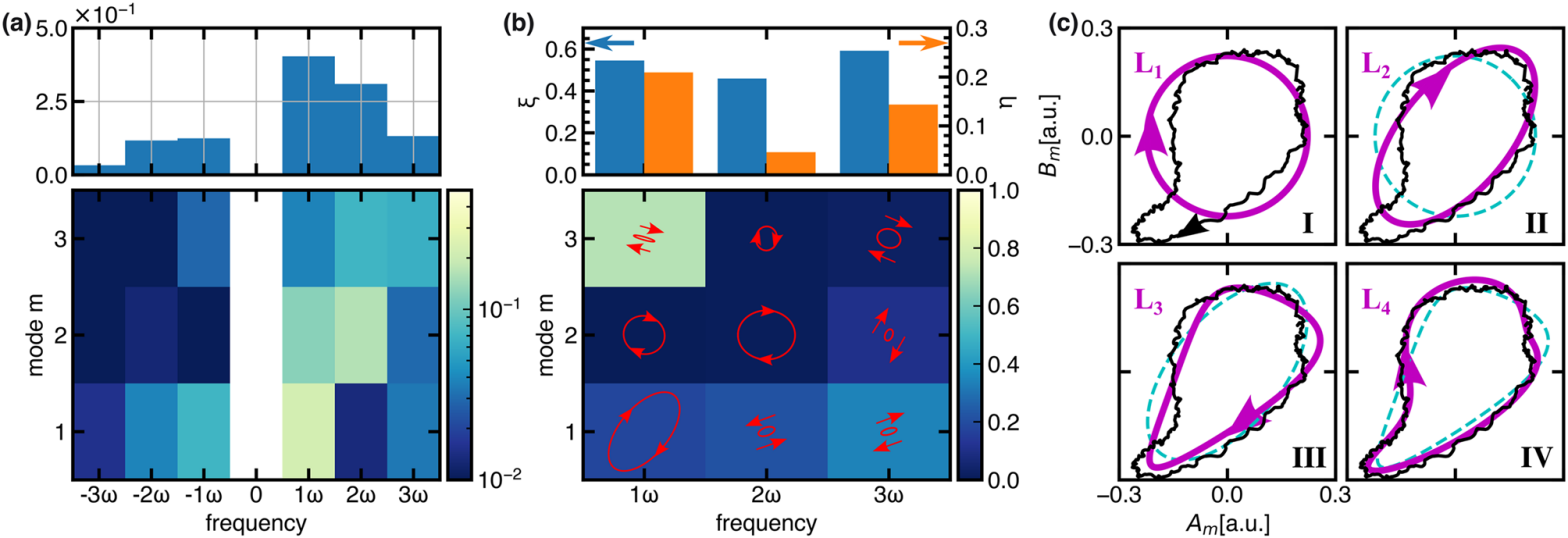
(**a**) Modal power obtained using the FBF, and the integrated modal power in terms of frequency (top). (**b**) The degrees of deformation $\xi$ and polarization $\eta$ as a function of frequency. The parameters are integrated in terms of azimuthal mode (upper). The polarization parameter $\gamma$ for each mode (lower) with the corresponding Lissajous trajectory (or polarization pattern). (**c**) An example of the deformation process of the *m *= 1 mode. (I) The circular Lissajous trajectory corresponds to the original harmonic mode, $\text {L}_{1}$, (magenta) and is shown with experimental data (black). (II) The deformed trajectory by superposition of counter-propagating modes of the fundamental frequency, $\text {L}_{2}$, (magenta) with the trajectory of the previous step (dashed cyan) for the reference. (III) and (IV) Further superposition of non-harmonic modes of $2\omega$ and $3\omega$ one by one, deforms the elliptical trajectory $\text {L}_{2}$ to the ones, $\text {L}_{3}$ and $\text {L}_{4}$. The final one is almost identical to the experimental data.

Consequently, the deformation can be further quantified by evaluating the power and polarization characteristics of each mode^32^. Here, the concept of “polarization” is used in analogy of electromagnetic field polarization of light in optics. Distinction between right- (electron diamagnetic) and left-handed (ion diamagnetic) rotation is made using the following Fourier transformation,

$$\begin{aligned} A_{\pm }(m, n\omega )&= \frac{1}{\sqrt{\pi T}}\int \tilde{\epsilon }(r,\theta ,t)\cos (m\theta \pm n\omega t)dSdt,\\ B_{\pm }(m, n\omega )&= \frac{1}{\sqrt{\pi T}}\int \tilde{\epsilon }(r,\theta ,t)\sin (m\theta \pm n\omega t)dSdt, \end{aligned}$$

where $A_{+}$, $B_{+}$, $A_{-}$ and $B_{-}$ represent the coefficients of right- and left-hand rotation, respectively, and the respective power can be expressed as $P_{i}(m, n\omega )=A_{i}^2+B_{i}^2 (i=+, -)$. Figure 4 shows the modal power $P(m, n\omega )$ obtained from the above transformation and the polarization characteristics resolved by frequency $n\omega$ and azimuthal mode number *m*, respectively. Then, the degree of the spatial pattern deformation of *M*-th harmonics is evaluated by a deformation index $\xi$, defined below,

$$\begin{aligned} \xi (M)=\frac{\sum _{m}P(m, M\omega )-P(M, M\omega )}{P(M)}, \end{aligned}$$

where $P(M)=\sum _{m}P(m, M\omega )$. The parameter indicates the non-harmonic mode contribution to the deformation of the *M*-th harmonic mode. On the other hand, emergence of backward propagating mode $(n<0)$ also changes the modal polarization property or causes the deformation of the Lissajous trajectories. A modal polarization parameter to indicate the linear polarization property, $\gamma$ , is introduced as,

$$\begin{aligned} \gamma (m, M>0)=\frac{P_{Linear}(m, M\omega )}{P_{Circular}(m, M\omega )+P_{Linear}(m, M\omega )}, \end{aligned}$$

where, $P_{Linear}(m, M\omega )=2min(P_{+}, P_{-})$ and $P_{Circular}(m, M\omega )=P_{+}+P_{-}-P_{Linear}$. The parameter is shown in under panel of Fig. 4b, which indicates that the Lissajous trajectory becomes more elliptical as the parameter, $\gamma$, becomes larger. Moreover, an index $\eta$, which represents the degree of linear polarization contamination, is defined as a function of frequency as follows,

$$\begin{aligned} \eta (M)=\frac{\sum _{m}P_{\pm }(m, M\omega )\gamma (m, M\omega )}{P(M)}, \end{aligned}$$

where $P_{\pm }(m, M\omega )=P(m, -M\omega )+P(m, M\omega )$. The deformation and polarization index, $\xi (M)$, $\eta (M)$, are shown in the upper panel of Fig. 4b. Moreover, the total deformation $\xi _{tot}$ and polarization $\eta _{tot}$ index are 0.525 and 0.146, respectively, whose defined as,

$$\begin{aligned} \xi _{tot}=\frac{\sum _{M}\xi (M)P(M)}{\sum _{M}P(M)}, \eta _{tot}=\frac{\sum _{M}\eta (M)P(M)}{\sum _{M}P(M)}. \end{aligned}$$

The analyses show that the deformation of the solitary oscillations is quantified by the contamination of non-harmonic azimuthal modes, which cause amplitude and phase modulation to the original harmonic oscillations, as well as modifying the modal polarization characteristics in the Lissajous trajectories.

Figure 4c illustrates an example to clarify the deformation process by non-harmonic modes for the case of $m=1$ mode. The circular trajectory shown in Fig. 4c-I is an original harmonic mode which is deformed into an elliptical trajectory as shown in Fig. 4c-II by the emergence of the counter-propagating modes or contamination of the linear polarization. Then, the emergence of non-harmonic modes of $2\omega$ or $3\omega$ one by one, deforms the elliptical trajectory is further to the trajectories shown in Fig. 4c-III, IV, finally resulting in a trajectory that is almost identical to the experimental data.

**Figure 5:**
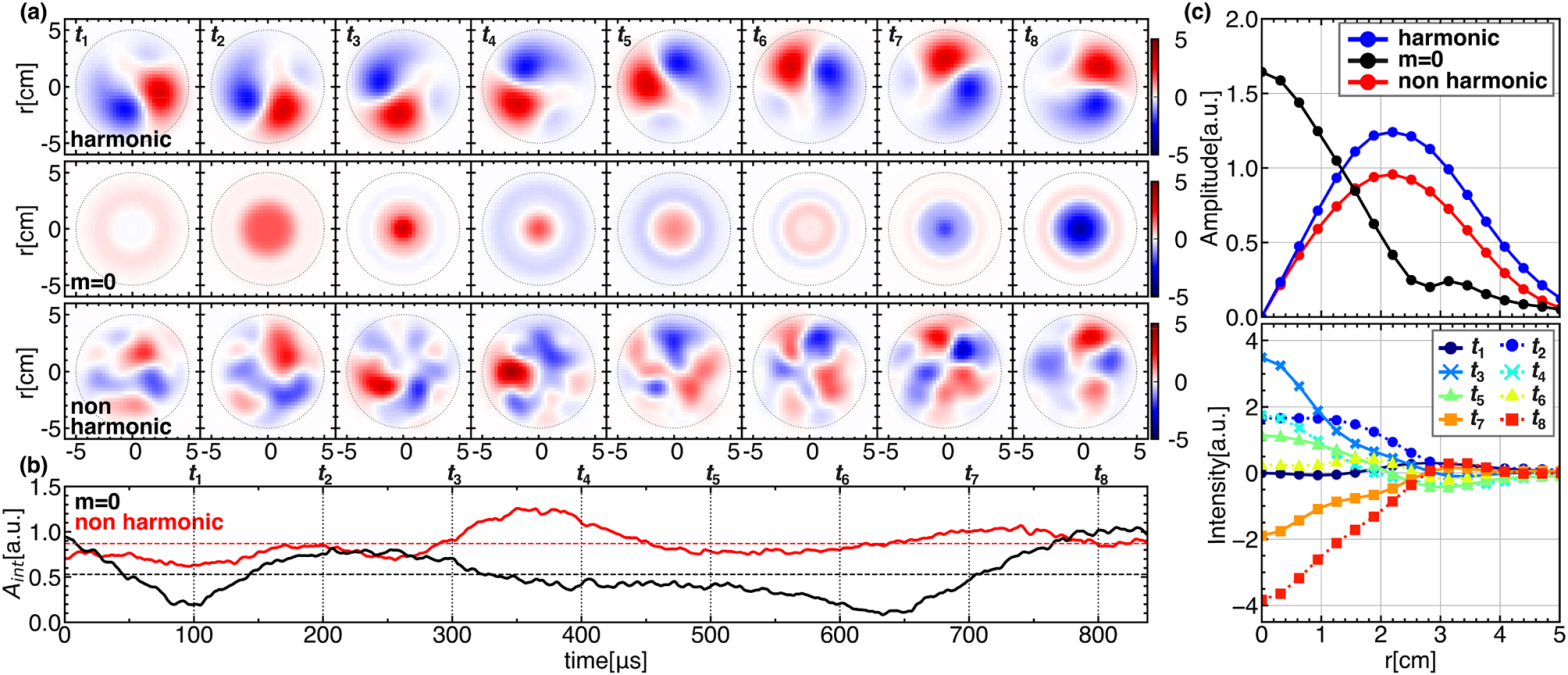
(**a**) The temporal evolutions of 2D images of inferred original solitary oscillations, generated *m *= 0 mode (symmetric) part, and integrated (asymmetric) part of non-harmonic modes. (**b**) The temporal evolutions of the integrated amplitudes of symmetric and asymmetric part. The dashed lines show the temporal average of each part. (**c**) The radial profiles of temporally averaged amplitudes of original, symmetric and asymmetric part (upper). The radial profiles of symmetric part as a function of time (lower).

## Discussion

A question to arise is how these non-harmonic modes should emerge. The most possible explanations should be that these modes should be produced with nonlinear interaction of the original harmonic modes with the background asymmetry structure composed of stational non-zero azimuthal modes, i.e., $\omega =0$. A simple sinusoidal algebra can give a mathematical base to non-harmonic mode production and polarization modification due to such nonlinear interaction. Supposed that background asymmetry, $\bar{\epsilon }_{A}\propto \cos (m\theta )$ and an original harmonic mode, $\tilde{\epsilon }_{h}\propto \cos (m'\theta +m'\omega t)$, their couplings following a simple algebra should produce the non-harmonic modes as,

$$\begin{aligned}{}&\bar{\epsilon }_{A}\cdot \tilde{\epsilon }_{h}\propto \cos (m\theta )\cos (m'\theta +m'\omega t)\\&\quad = \cos ((m+m')\theta +m'\omega t)+\cos ((m-m')\theta -m'\omega t). \end{aligned}$$

The nonlinear coupling should produce two modes whose azimuthal mode numbers are $m+m'$ and $m-m'$ . Moreover, if the integer, *m*, is larger than $m'$, the mode of the second term propagates in the opposite direction to the original mode. For example, the dominant harmonic mode of $m=1$ should produce backward propagating mode through the couplings with the stationary $m=2$ structure of the background. Similarly, all non-harmonic modes observed here can be produced through the nonlinear couplings between the original harmonic modes of $1\omega , 2\omega , 3\omega \ldots$, and the background asymmetry of the corresponding azimuthal mode numbers. Note that the harmonic modes are assumed to be self-produced through their nonlinear couplings. The presented nonlinear coupling process between the stationary structure $(\omega =0)$ and the fluctuating modes $(\omega \ne 0)$ should be a different kind of the ones between fluctuating modes, which have been conventionally discussed^33–35^.

Without background asymmetry, the solitary oscillations should consist only of simple harmonic components. The hypothesis makes it possible to reconstruct the original solitary oscillation composed of the original harmonic modes. The produced non-harmonic modes also include symmetric structure ($m=0$ mode) in the case of $m=m'$, in addition to asymmetric structure ($m\ne 0$ modes) that deforms the spatial pattern. Figure 5 shows the three temporal evolutions of the tomographic images: the original solitary oscillations, and the produced $m=0$ mode, and the $m\ne 0$ mode reconstructed from non-harmonic modes, with their global amplitudes and temporally averaged radial profiles: here the reconstruction of original solitary oscillations uses the azimuthal mode of $m=1, 2$ and 3.

From the images, the $m\ne 0$ (asymmetric) mode exhibits complex deformations, whereas the $m=0$ (symmetric) mode shows large variations in the core region of the plasma. Moreover, since the amplitudes of symmetric and asymmetric modes are of the same order, the coupling should not only deform the harmonic mode to make the oscillation pattern more turbulent, but also recover the symmetry. Moreover, in $m=0$ mode, the temporally averaged radial profile of amplitude shows a steep gradient change at $r\sim 3$ cm in Fig. 5c. If the emission is proportional to the pressure of the plasma, the steep gradient could correspond to a shear creation in the diamagnetic velocity. It is possible that such shear creation could affect the instabilities^36–40^. Moreover, a resent simulation has suggested that nonlinearity interaction between the the background density asymmetry caused by inhomogeneous particle sources and the drift wave can enhance zonal flows, which may be consistent with the present observation^41^. The present scenario leads to a hypothesis that steady state asymmetries in magnetically confined plasma, which could be induced by auxiliary heating, may enhance turbulence or zonal flows to change the confinement properties.

Finally, the measurements and analyses on the plasma images obtained with the tomography show that the nonlinear effects of background asymmetry, its nonlinear coupling with the harmonic modes, should produce the non-harmonic modes that deform the original pattern of the solitary oscillation with breaking the rotational symmetry. The results suggest an unfeatured route for plasma to change into turbulence state, as well as the excellent capability of the tomography to explore a research frontier for magnetized plasmas.

## Methods

### Plasma assembly for nonlinear turbulence analysis (PANTA)

PANTA is a linear magnetized plasma device that generates cylindrical plasma 100 mm in diameter and 4000 mm long by RF heating. The vacuum chamber is 450 mm in diameter and 4050 mm long, and is modularized into 16 vacuum vessels, allowing for flexible selection of the location of the measurement system. The axial magnetic field is generated by 17 coils installed around the vacuum vessel, and the field strength can be flexibly set from 0.01 to 0.15 T according to the experiment. Neutral gas (Argon) is filled from near the heating source of the plasma. The filling gas pressure is controlled by adjusting the flow rate with a mass flow meter. Five turbo molecular pumps installed at the rear of PANTA exhaust air at 1400 L/s to maintain a high vacuum. In the plasma heating source, The helicon wave at 7 MHz is excited from a copper double-loop antenna wound around a 100 mm diameter quartz tube. The input power can be flexibly set from 3.0 to 6.0 kW.

### Tomography system and reconstruction algorithm

The 4 light-guide array tomography system is installed in PANTA, in which 4 pairs of light-guide arrays are arranged at 45-degree intervals in the azimuthal direction of the plasma. The tomography system has a total of 128 channels of measurement line of sight, and detects ArI emission signals by passing optical filters. The emission signal is recorded as a voltage signal by a current-voltage converter. The measurement line-of-sight covers the plasma and is equally spaced within $\pm 80$ mm from the center of the device (plasma center). The local emission of the plasma is reconstructed using a reconstruction algorithm called the Maximum Likelihood-Expectation Maximization method^26^ to display a 160 mm $\times$ 160 mm observation range in an $11\times 11$ grid with a spatial resolution of $\sim 1.45$ cm, which is comparable to the Larmor radius of the PANTA plasma. This method is suitable for reconstruction of plasma structures that are discrete and abruptly changing both temporally and spatially.

## Data Availability

The data analyzed in this study are available from the corresponding author on reasonable request.
